# Brewhouse-Resident Microbiota Are Responsible for Multi-Stage Fermentation of American Coolship Ale

**DOI:** 10.1371/journal.pone.0035507

**Published:** 2012-04-18

**Authors:** Nicholas A. Bokulich, Charles W. Bamforth, David A. Mills

**Affiliations:** 1 Department of Viticulture and Enology, Robert Mondavi Institute of Wine and Food Science, University of California Davis, Davis, California, United States of America; 2 Department of Food Science and Technology, Robert Mondavi Institute of Wine and Food Science, University of California Davis, Davis, California, United States of America; University of Nottingham, United Kingdom

## Abstract

American coolship ale (ACA) is a type of spontaneously fermented beer that employs production methods similar to traditional Belgian lambic. In spite of its growing popularity in the American craft-brewing sector, the fermentation microbiology of ACA has not been previously described, and thus the interface between production methodology and microbial community structure is unexplored. Using terminal restriction fragment length polymorphism (TRFLP), barcoded amplicon sequencing (BAS), quantitative PCR (qPCR) and culture-dependent analysis, ACA fermentations were shown to follow a consistent fermentation progression, initially dominated by *Enterobacteriaceae* and a range of oxidative yeasts in the first month, then ceding to *Saccharomyces* spp. and *Lactobacillales* for the following year. After one year of fermentation, *Brettanomyces bruxellensis* was the dominant yeast population (occasionally accompanied by minor populations of *Candida* spp., *Pichia* spp., and other yeasts) and *Lactobacillales* remained dominant, though various aerobic bacteria became more prevalent. This work demonstrates that ACA exhibits a conserved core microbial succession in absence of inoculation, supporting the role of a resident brewhouse microbiota. These findings establish this core microbial profile of spontaneous beer fermentations as a target for production control points and quality standards for these beers.

## Introduction

American coolship ale (ACA) is a type of beer produced in the United States using production practices adopted from the lambic brewers of Belgium, in an attempt to create a similar style of sour ale. Traditional lambic is fermented entirely spontaneously by exposing the cooling, boiled wort to the atmosphere overnight in an open, shallow vessel known as a “coolship,” during which time it becomes inoculated by autochthonous yeasts and bacteria that perform the fermentation, lasting 1–3 years in oak casks [1], [2]. During this time, several stages of succession occur in the microbial community profile that are likely responsible for the unique flavors exhibited by these beers. Previous culture-based studies revealed that *Enterobacteriaceae* and oxidative yeasts (*Kloeckera* spp.) dominate the early fermentation, lasting 1–2 months, after which *Saccharomyces* spp. take hold and are responsible for the main alcoholic fermentation and *Pediococcus* spp. proliferate, producing copious quantities of lactic acid [1], [3]. Finally, *Brettanomyces* spp. dominate the late stage of fermentation after 1 year, producing volatile phenols and other characteristic aroma compounds [3]. The combined metabolic activity of several microbial populations, the low pH, and the high ethanol environment result in the production of very high concentrations of aromatic esters, volatile phenols, and other compounds responsible for the unique sensory qualities of these beers [3], [4]. ACA, currently produced by a small number of breweries, is a direct adaption by American craft brewers to recreate a lambic-style beer, adopting the same recipes and production methods employed by the Belgian lambic breweries. However, to our knowledge, neither of these beers has ever been studied using culture-independent techniques, and how geographical location influences this fermentation microbial profile has yet to be determined.

In this study, ACAs from different batches were selected from one American brewery and followed using TRFLP to give a low-resolution view of bacterial community patterns as well as high-resolution taxonomic identification of yeasts and lactic acid bacteria (LAB) across samples and across time. Representative samples were then selected from clusters identified by TRFLP and sequenced using 16S rDNA barcoded amplicon sequencing (BAS) on the Illumina GAIIx to provide an in-depth look at bacterial community structure over time. Quantitative PCR (qPCR) and culture-based techniques were also used to characterize changes in the microbial community over time. Findings established that ACA fermentation involves a multiphase, core microbial profile, which is conserved batch-to-batch, supporting the presence of resident brewhouse microbiota responsible for conducting the fermentation. Additionally, this core profile displayed some notable similarities to the microbial profile of lambic, suggesting that the shared production methods exert a common selective niche environment for spontaneous beer fermentation.

## Materials and Methods

### Sampling

Wort was prepared according to the standard protocol of the brewery studied, with a typical mash followed by at least 1 hr of kettle boiling, after which wort was transferred to an open coolship and left to cool overnight. The following morning, after the wort reached ∼22°C, it was transferred to enclosed oak barrels and fermented at cellar temperature. Barrels were topped off as needed to minimize headspace exposure to air during fermentation. Prior to bottling, a fruit slurry was added to the beer and allowed to referment in bottles.

**Figure 1:**
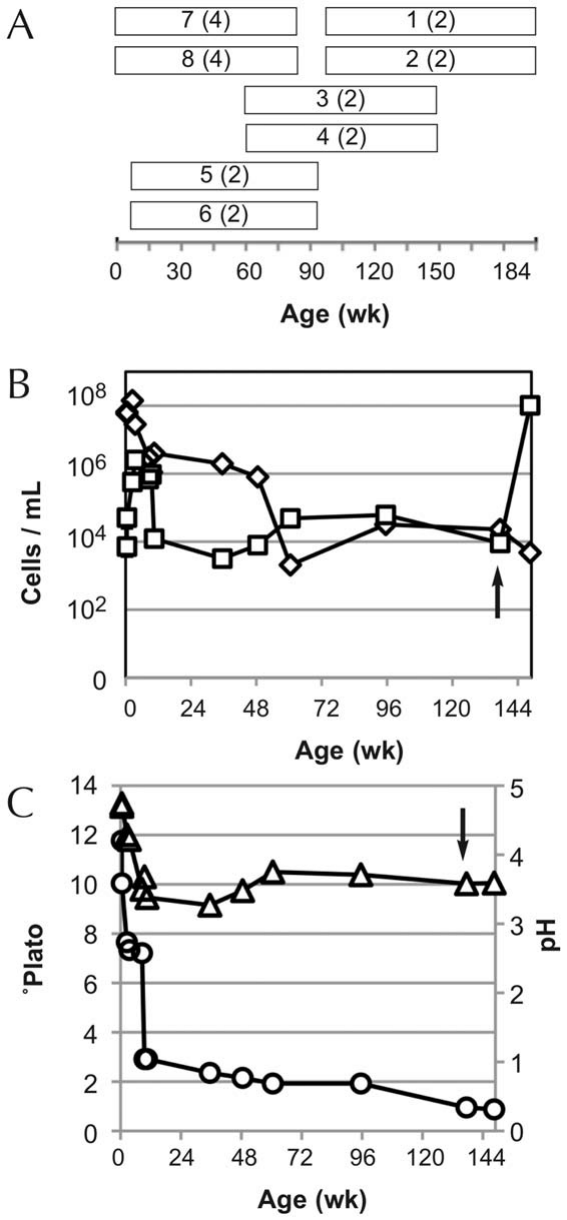
ACA fermentation profile and sampling regime. *Panel A*: Sampling regime employed representing 3 years of ACA fermentation. White bars represent the span of sampling times for each batch. Labels indicate batch number (number of barrel replicates in parenthesis). *Panels B/C*: Real-time PCR of total bacteria and total yeast populations (*Panel B*) and pH and °Plato (*Panel C*) across ACA fermentation. All values are averages of multiple batches tested in duplicate (where possible; Figure S1). Error bars indicate ±1 standard deviation. ◊, Total bacteria; □, total yeasts; ▵, pH; ○, °Plato. Arrow indicates time at which fruit was added and beer was refermented in bottle.

Samples were collected starting the morning after overnight exposure of the wort to the atmosphere in the coolship (wk 0) through up to 184 wk of fermentation in oak casks (Figure 1A; Figure S1). Some bottle-refermented samples were also tested from batches one and two (wk 148). In order to collect samples representing the complete 3-yr fermentation of ACA, eight different batches were sampled representing an overlapping mosaic of time points (Figure S1): batches one and two covered wk 95–184; batches three and four from wk 60–149; batches five and six from wk 8–92; and batches seven and eight from wk 0–83. Replicate barrels from each batch were sampled, where possible, and the same barrels were followed throughout the sampling period (Figure 1A; Figure S1). Samples of fresh wort from batches seven and eight were also collected prior to coolship exposure to ensure that populations detected by culture-independent methods were not due to residual DNA from grain-associated populations surviving kettle boil; no amplification could be achieved from any of these samples.

Samples were collected aseptically from sample ports located in the head of the barrels, 5–6 in. above the lower rim. Approximately 200 mL were bled from the sampling port before sample collection. Fresh samples used for culture-dependent techniques were rush-shipped on ice, stored at 4°C until analysis, and processed within 24 hr. Samples for TRFLP analysis only were immediately (within 24 hr of sampling, immediately following shipping on ice) centrifuged at 4,000× g for 15 min at 4°C, decanted, and the remaining pellet stored at −20°C until analysis.

### DNA Extraction

From the centrifuged samples, 100 µL of cell pellet were removed and washed 3 times by suspension in 1 mL ice-cold PBS, centrifugation at 8,000× g (5 min), and the supernatant discarded. The cell pellet was then suspended in 200 µL DNeasy lysis buffer (20 mM Tris-Cl [pH 8.0], 2 mM Sodium EDTA, 1.2% Triton X-100) supplemented with 40 mg/mL lysozyme and incubated at 37°C for 30 min. From this point, the extraction proceeded following the protocol of the Qiagen Fecal DNA Extraction Kit (Qiagen, Valencia, CA), with the addition of a bead beater cell lysis step of 2 min at maximum speed following addition of “buffer ASL” using a FastPrep-24 bead beater (MP Bio, Solon, OH). DNA extracts were stored at −20°C until further analysis. Duplicate extractions were made for all samples, except for those samples (very old samples still maturing in barrels) containing such low concentrations of cells that the entire sample needed to be used for one extraction to obtain a workable quantity of DNA.

### TRFLP Analysis

PCR amplification was performed in 50-µL reactions containing 1 µL of DNA template, 25 µL 2× Promega GoTaq Green Master Mix (Promega, Madison, WI), 1 mM MgCl_2_, and 2 pmol of each primer. Each PCR was performed in triplicate and the products combined prior to purification.

For amplification of the ITS1/ITS4 domain of yeast 26S rDNA genes (ITS-TRFLP) [5], the forward primer used was ITS1HEX (5′-[5HEX] TCCGTAGGTGAACCTGCGG-3′) and the reverse primer was ITS4 (5′-TCCTCCGCTTATTGATATGC-3′) [6]. The PCR conditions were an initial denaturation at 95°C for 2 min, followed by 30 cycles of denaturation at 95°C for 1 min, annealing at 50°C for 1 min, and extension at 72°C for 2 min, and with a final extension at 72°C for 7 min.

For amplification of universal bacterial 16S rDNA genes (16S-TRFLP), the forward primer used was Uni331F-FAM (5′-[5FAM] TCCTACGGGAGGCAGCAGT-3′) [7] and the reverse primer was 1492R (5′-GGTTACCTTGTTACGACTT-3′) [8]. The PCR conditions were an initial denaturation at 95°C for 2 min, followed by 30 cycles of denaturation at 95°C for 30 sec, annealing at 50°C for 30 sec, and extension at 72°C for 2 min, and with a final extension at 72°C for 5 min.

Lactic acid bacteria (LAB)-specific TRFLP (LAB-TRFLP) [9] was performed using the primers NLAB2F (5′-[5HEX]-GGCGGCGTGCCTAATACATGCAAGT-3′) and WLAB1R (5′-TCGCTTTACGCCCAATAAATCCGGA-3′) [9]. PCR conditions consisted of an initial denaturation at 95°C for 5 min, followed by 30 cycles of denaturation at 95°C for 45 sec, annealing at 66°C for 30 sec, and extension at 72°C for 45 sec, and with a final extension at 72°C for 5 min.

All samples were amplified in triplicate and combined prior to purification using QIAquick PCR Purification Kit (Qiagen), following the manufacturer's instructions. Restriction digests were performed according to the manufacturer's instructions for each individual enzyme. Digestions of ITS PCR products were performed using HaeIII, DdeI, and HinfI. Digestions of 16S PCR products were performed using AluI, MspI, HaeIII, and HhaI. Digestions of LAB-TRFLP products used MseI and Hpy118I. The digested DNA was submitted to the UC Davis College of Biological Sciences Sequencing Facility, for fragment separation via capillary electrophoresis. Traces were visualized using the program Peak Scanner v1.0 (Applied Biosystems, Carlsbad, CA) using a baseline detection value of 10 fluorescence units. Peak filtration and clustering were performed with R software using the scripts and analysis protocols designed by Abdo and colleagues [10]. Operational taxonomic unit (OTU) picking for both LAB-TRFLP and 16S-TRFLP was based on *in silico* digest databases generated by the virtual digest tool from MiCA [11] of good-quality 16S rDNA gene sequences compiled by the Ribosomal Database Project Release 10 [12], [13], allowing up to 3 nucleotide mismatches within 15 bp of the 5′ terminus of the forward primer. Putative species assignments of ITS sequence fragments were made by comparing fragment peak data to a Wine Yeast ITS TRFLP database developed in-house [5]. Principal coordinates were computed from Bray-Curtis dissimilarity scores of raw TRFLP data (prior to taxonomic classification/grouping) using QIIME [14].

### Illumina Sequencing Library Construction

For amplification of the V4 domain of bacterial 16S rRNA genes, we used primers F515 and R806 [15], both modified to contain an illumina adapter region for sequencing on the illumina GAIIx platform and, on the forward primer, an 8 bp Hamming error-correcting barcode to enable sample multiplexing [16]. A list of V4 primers and barcodes used is presented in Table S1. PCR reactions contained 5–100 ng DNA template, 1× GoTaq Green Master Mix (Promega), 1 mM MgCl_2_, and 2 pmol of each primer. Reaction conditions consisted of an initial 94°C for 3 min followed by 35 cycles of 94°C for 45 sec, 50°C for 60 sec, and 72°C for 90 sec, and a final extension of 72°C for 10 min. All samples were amplified in triplicate and combined prior to purification. Amplicons were purified using the Qiaquick 96 kit (Qiagen), quantified using PicoGreen dsDNA reagent (Invitrogen, Grand Island, NY), mixed at equimolar concentrations, and gel-purified using the Qiaquick gel extraction kit (Qiagen) all according to respective manufacturers' instructions. Purified libraries were submitted to the UC Davis Genome Center DNA Technologies Core for cluster generation and 150 bp paired-end sequencing on the Illumina GAIIx platform.

### Data Analysis

Raw Illumina fastq files were demultiplexed, quality-filtered, and analyzed using QIIME [14]. The 150-nt reads were truncated at any base receiving a quality score <1e-5, and any read containing one or more ambiguous base call was discarded, as were truncated reads of <75 nt. OTUs were assigned using the QIIME implementation of UCLUST [17], with a threshold of 97% pairwise identity, and representative sequences from each OTU selected for taxonomy assignment. OTUs were classified taxonomically using a QIIME-based wrapper of the Ribosomal Database Project (RDP) classifier [18] against the RDP 16S rDNA database core set [12], [13], using a 0.80 confidence threshold for taxonomic assignment. Any OTU representing less than 0.01% of the total sequences was removed to avoid inclusion of erroneous reads, leading to inflated estimates of diversity. Filtered sequences were aligned using PyNast against a template alignment of the RDP core set filtered at 97% similarity. Beta diversity estimates were calculated within QIIME using weighted UniFrac [19] distances between samples. From these estimates, principal coordinates were computed to compress dimensionality intro three-dimensional principal coordinate analysis (PCoA) plots. Observed-species alpha-rarefaction of filtered OTU tables was also performed in QIIME to confirm that sequence coverage was adequate to capture the species diversity observed in all samples (Figure S2).

### Quantitative PCR (qPCR)

Quantitative PCR was performed in 20-µL reactions containing 10–100 ng of DNA template, 0.2 µM of each respective primer, and 10 µL of Takara SYBR 2× Perfect Real Time Master Mix (Takara Bio Inc). For amplification of total bacteria, the primers Uni334F (5′-ACTCCTACGGGAGGCAGCAGT-3′) [20] and Uni514R (5′-ATTACCGCGGCTGCTGGC-3′) [21] were used. Reaction conditions included an initial hold at 95°C for 20 sec, followed by 40 cycles of 4 sec at 95°C and 25 sec at 65.5°C. For amplification of total yeast, the primers YEASTF (5′-GAGTCGAGTTGTTTGGGAATGC-3′) and YEASTR (5′-TCTCTTTCCAAAGTTCTTTTCATCTT-3′), producing a 124-bp fragment, were used [22]. Reaction conditions involved an initial step at 95°C for 10 min, followed by 40 cycles of 15 sec at 95°C, 1 min at 60°C, and 30 sec at 72°C. Cell concentration was calculated by comparing sample threshold values (*C_T_*) to a standard curve of *S. cerevisiae* or *Escherichia coli* genomic DNA extracted from known cell concentrations. All reactions were performed in triplicate in optical-grade 96-well plates on an ABI Prism 7500 Fast Quantitative PCR System (Applied Biosystems). The instrument automatically calculated cycle threshold (*C_T_*), efficiency (*E*), and confidence intervals. Melt curve analysis was performed after thermal cycle program completion for both assays to assess the specificities of the amplicons.

### Culture-dependent Identification

Fresh samples were serially diluted in 1% saline buffer and plated on Mann-Ragosa-Sharp Agar (MRS) supplemented with 25 mg/L cycloheximide (for the selective detection of lactic acid bacteria) and Wallerstein Differential Agar (WLD) supplemented with 1% (wt/wt) Sodium Bicarbonate and 25 mg/L cyclohexamide (for the differential detection of bacteria). All plates were incubated aerobically at 25°C for 3–7 days and then colonies were counted and recorded. Morphologically distinct colonies growing on differential agar were isolated by restreaking three consecutive times on plate count agar. Two isolates were obtained for each colony morphotype. Single colonies of pure isolates growing on plate count agar were removed and suspended in 20 µL GeneReleaser (BioVentures, Inc., Murfreesboro, TN). This suspension was microwaved at medium power for 10 min prior to addition of PCR reagents without mixing the contents of the tube. PCR amplification was performed in 50-µL reactions containing 25 µL 2× Promega GoTaq Green Master Mix (Promega), 1 mM MgCl_2_, and 0.2 µL of each primer. Bacterial 16S rDNA gene amplification was performed using the forward primer 27F (5′-AGAGTTTGATCCTGGCTCAG-3′) and reverse primer 1492R (5′-GGTTACCTTGTTACGACTT-3′) [8]. The thermocycler program consisted of a denaturation at 94°C for 10 min; followed by 35 cycles of a denaturation at 94°C for 1 min, annealing at 50°C for 1 min, and an extension at 72°C for 1.5 min, and a final extension step of 72°C for 10 min. All positive amplicons were purified using QIAquick PCR Purification Kit (Qiagen), following the manufacturer's instructions, and submitted to the UC Davis College of Biological Sciences Sequencing Facility for sequencing using the same primers. Sequence construction was performed using 4 peaks (www.mekentosj.com/science/4peaks) and alignment using NCBI BLAST.

## Results

All batches were brewed in the Spring and Winter of 2008, 2009, and 2010 in an independent craft brewery located in the Northeastern United States. Following wort production, boiled wort was pumped into the brewery's stainless steel coolship, located in a separate portion of the facility from the brewhouse and cellar, and allowed to cool overnight under circulating air. The following morning, once cooled to approximately 22°C, the wort was pumped into barrels and left to ferment at room temperature. Fermentation typically began within one week (as indicated by gas formation). Samples were collected starting in the coolship and continuing throughout the fermentation at regular intervals (Figure 1A; Figure S1). In addition to tracking bulk microbial composition using qPCR (Figure 1B), pH and °Plato were measured to track the rate of fermentation (Figure 1C).

### Quantitative PCR of ACA fermentations

Quantitative PCR (qPCR) was used to quantify total yeasts and bacteria during ACA fermentation (Figure 1B). As TRFLP and Illumina sequencing provide relative community structure data but no indication of absolute abundance, this step was necessary to compare differences among time points. Total bacterial load was initially high in all batches, with an average of 6.5×10^7^ cells/mL (±1.2×10^6^) at wk 0 and growing steadily to 1.4×10^8^ cells/mL (±7.9×10^6^) at wk 2 (Figure 1B). Counts then steadily declined in all samples after wk 2 (coordinately with the decrease in pH and °Plato; Figure 1C), reaching 2.1×10^3^ cells/mL (±2.0×10^2^) at wk 60. From here, the average populations gradually regenerated, reaching 2.3×10^4^ cells/mL (±1.1×10^3^) at wk 137; average populations were 4.8×10^3^ cells/mL (±9.4×10^1^) at wk 148 in bottled, refermenting ACA.

The average total yeast population quickly grew from 7.2×10^3^ cells/mL (±3.6×10^3^) cells/mL at wk 0, peaking at 2.6×10^6^ cells/mL (±4.1×10^5^) at wk 4. Populations remained near this level through wk 9 but quickly dropped to 2.2×10^4^ cells/mL (±4.0×10^3^) at wk 11 (following ∼80% attenuation of soluble solids as °Plato); average total yeast populations reached a minimum of 3.2×10^3^ cells/mL (±2.0×10^2^) at wk 36 before regrowing into the 10^4^ range from wk 60 onward. After bottle refermentation on fruit, the average total yeast population reached 1.0×10^8^ cells/mL (±1.4×10^7^) in all bottles.

### Yeast community structure

**Figure 2:**
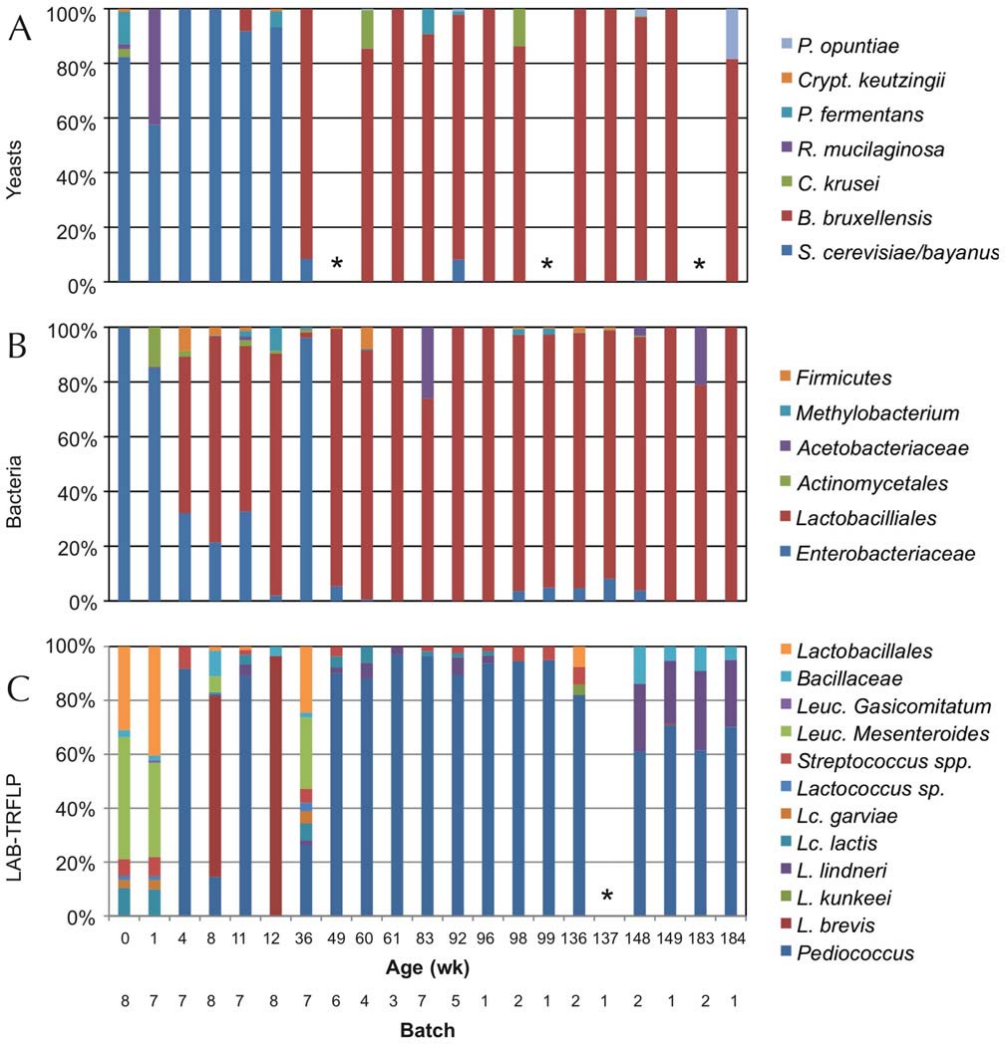
TRFLP analysis of ACA fermentation succession across complete timescale. Normalized relative OTU peak areas for multiple ACA fermentations observed over a 3-year period by ITS-TRFLP (yeasts, *Panel A*), 16S-TRFLP (bacteria, *Panel B*), and LAB-TRFLP (lactic acid bacteria, *Panel C*). All samples were tested in duplicate, when possible, and each bar represents averaged duplicates for a single time point, single barrel. *y*-axes indicate relative OTU abundance. *Sample was not amplifiable with these specific primers.

In lambic, a diverse range of yeasts are present, but *B. bruxellensis* is the most dominant yeast and exerts a significant impact on the aroma [1]. Therefore, ITS-TRFLP [5] was used to describe ACA yeast diversity over time and to determine whether ACA fermentation displays a similar yeast community to lambic. Initially, all fermentations displayed relatively high yeast diversity, but the dominant population was already 60–80% (relative abundance) *S. cerevisiae* from wk 0 (Figure 2A). Other yeasts detected at wk 0 in different samples include: *Candida krusei*, *Pichia fermentans/kluyveri*, *Cryptococcus keutzingii*, and *Rhodotorula mucilaginosa*. By wk 1, all batches demonstrated a shift to ∼60% *S. cerevisiae* and ∼40% *R. mucilaginosa*. By wk 4, all fermentations were homogeneously *S. cerevisiae* until wk 11, when *B. bruxellensis* first appeared in some of the fermentations (average relative abundance 7%). As of wk 36, all fermentations were dominated by *B. bruxellensis* with an occasional trace of *S. cerevisiae* (including at wk 148, in a bottled, refermenting beer). *B. bruxellensis* remained the dominant yeast (64–100% relative abundance) until the end of fermentation and after bottling, with minor populations of *P. opuntiae*, *P. fermentans/kluyveri*, C. *keutzingii*, and *C. krusei* cropping up intermittently.

### Bacterial community structure

TRFLP was used to track bacterial community structure in ACA over 3 yr of fermentation. In lambic, *Pediococcus* and enterobacteria play prominent parts in acidifying the beer and producing fatty acids related to characteristic aroma development, respectively [1]. Thus, total bacterial community structure was queried using 16S-TRFLP in order to assess what bacteria are present throughout ACA fermentation and followed by LAB-TRFLP [9] to further dissect the substructure of *Lactobacillales*. TRFLP indicated that the early fermentation (wk 0–4) of ACA was consistently dominated by enterobacteria (Figure 2B). Plating identified the primary enterobacteria involved to be predominantly *Klebsiella oxytoca* and *Enterobacter agglomerans* but also *Enterobacter ludwigii*, *Enterobacter cloacae*, *Enterobacter mori*, *Klebsiella pneumonia*, and *Serratia ureilytica* at different times over the first 12 wk (Table 1). None were cultured after this time point, though traces of enterobacteria were detected at later time points in some batches by molecular methods.

**Table 1:** Taxonomic Assignments of Bacteria Isolated from ACA.

Closest Match	Accession #	Total Score	Max ID	Colony Morphotype	Weeks Detected*
*Paenibacillus provencensis* strain 4401170	EF212893.1	1916	95%	Sm., creamy, translucent, slightly glossy	1
*Enterobacter ludwigii* isolate PSB2	HQ242715.1	1977	97%	Med., creamy, rd., glossy, slimy	1–4
*Enterobacter mori* strain R3-3 16S	GQ406569.1	1977	97%	Med-lg., creamy, opaque, matte, smooth	1–4
*Serratia ureilytica* isolate PSB22	HQ242735.1	2097	98%	Sm., wh., opaque, rd.	1–4
*Pectobacterium carotovorum* subsp. *carotovorum* strain Ecc8-5 16S	FJ527484.1	2049	97%	Med., rd., creamy, glossy	1–8
*Klebsiella pneumoniae* strain 27F 16S	GU327663.1	2056	97%	Med., hazy, copious slimy yellow coating	1–8
*Enterobacter cloacae*	EF120473.1	1982	96%	Tiny, hazy, translucent, glossy	1–8
*Enterobacter hormaechei* strain M.D.NA5-9	JF690889.1	1995	96%	Med., rd., creamy, glossy	1–8
*Enterobacter agglomerans* strain A84	AF130948.2	1991	96%	Lg., snotty, opaque, yellow	1–12
*Klebsiella oxytoca* strain SHD-1	GU361112.1	2032	98%	Med., rd., creamy, glossy	1–12
*Lactobacillus brevis* strain b4	FJ227317.1	2073	98%	Med., opaque qhite, rough margin	8–148
*Acetobacter fabarum* strain NM118-1	HM218478.1	2043	98%	Med., rd., translucent orange, spreading	8–148
*Acetobacter lovaniensis* strain KS1	FJ157228.1	2074	98%	Med., hazy/translucent, glossy, smooth	1–148

* Indicates the time points (wk) at which colonies matching the morphotype of this organism were cultured. All colony morphotypes were isolated in duplicate for sequencing and identification.

By wk 4, *Lactobacillales* succeeded as the dominant population in all fermentations, their relative population steadily growing from 50–70% (wk 4–12) to >90% (wk 12) for the remainder of the fermentation (Figure 2B). The only LAB cultured was *Lactobacillus brevis* (Table 1), leading to the initial assumption that this was the dominant LAB in all batches. However, LAB-TRFLP was used to resolve populations of LAB more deeply, revealing a more intricate tapestry of LAB involved throughout the fermentation, and larger differences across batches than observed using 16S-TRFLP (Figure 2C). A rich diversity was observed in the first 2 wk, dominated by *Leuconostoc* spp. and with measurable contingents of *Lactococcus lactis*, *Lactococcus garviae*, *Streptococcus* sp., *Lactobacillus delbreuckii*, *Lactobacillus curvatus*, *L. brevis*, and *Lactobacillus kunkeei*. By wk 4, the LAB population was predominantly *Pediococcus* (>80%), with minor detection of *Lactobacillus fermentum*, *L. brevis*, *L. kunkeei*, and *Lc. lactis*. In the very late fermentation of several batches, *Lactobacillus lindneri* emerged as a prevalent minority population. As *Bacilli* are also well covered by the LAB-TRFLP protocol used [9], small populations of *Bacillales* (typically <5%, except for batch eight) were detected in different batches throughout the fermentation, as was *Paenibacillus* in the first 1–2 wk of some batches. A bacterium identified as *Paenibacillus provenciensis* was isolated at wk 1 from batch eight (Table 1), but was not detected in any batches thereafter (by culturing or TRFLP).

**Figure 3:**
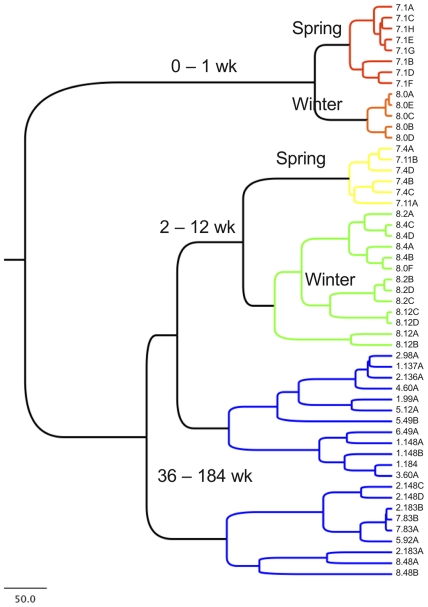
UPGMA hierarchical clustering demonstrates age-based grouping of samples. Hierarchical relationship among samples based on Euclidean distance of 16S-TRFLP OTU abundance profiles derived from the MspI restriction digest. Node labels (and associated colors) indicate age- and batch-based groups. Tip labels indicate batch number.wk.

In order to reveal relationships among samples based on community structure and to select representatives for BAS, samples were grouped via UPGMA hierarchical clustering based on Euclidean distance of MspI 16S-TRF profiles (Figure 3). Three distinct clusters form, dividing early (wk 0–1), middle (wk 2–12), and late fermentation (wk 36–184) samples, with the early- and middle-fermentation clusters each clearly bifurcating into Winter (batch eight) and Spring (batch seven) groups.

Therefore, 16 samples were selected from this set for Illumina sequencing, representing all defined clusters (at least 3 selected per cluster, with two independent samples representing each time point), all batches, and the complete time course of the fermentation, with the early fermentation highly sampled from batches seven and eight to further probe batch-to-batch variation.

### Barcoded Amplicon Sequencing

**Figure 4:**
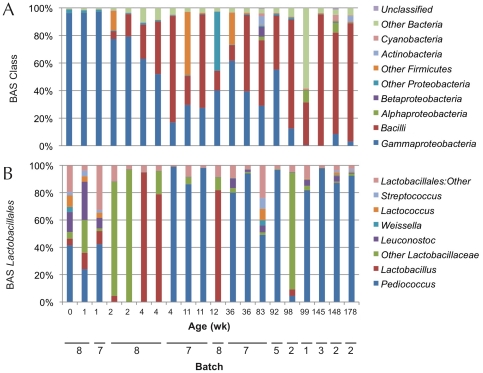
Bacterial taxa abundance measured using BAS 5′ sequences. *Panel A*: Class-level taxon relative abundance per sample as a percentage of total sequences. *Panel B*: Genus-level relative abundance of *Lactobacillales*. All bars represent a single sample from a single batch, and most time points are represented by at least two independent samples (where available), as presented. *y*-axes indicate relative OTU abundance.

As TRFLP is based on restriction mapping rather than true sequence data, BAS of universal 16S rDNA amplicons was applied to a representative subset of ACA to confirm taxonomic classifications, to identify low-abundance populations, and to perform phylogeny-based beta-diversity comparisons. Bacterial community profiles recovered by BAS revealed similar structures at the order level and genus level, compared to those observed by 16S-TRFLP and LAB-TRFLP, respectively (Figure 4). These data demonstrate the same general fermentation profile, initially dominated by *Enterobacteriaceae* and quickly succeeded by *Lactobacillales*, though by this method enterobacteria were detected at higher levels longer into the fermentation. Unfortunately, BAS could not differentiate the majority of *Enterobacteriaceae* beyond the family level (same level as TRFLP), so we must still rely on the culturing data to provide species identification of enterobacteria present. As no enterobacteria could be cultured beyond wk 12, it is unclear whether this population is experiencing its own internal species succession, or whether this is nonviable or non-active artifact from the group observed in the early fermentation.

At the genus level, the LAB community structure uncovered by BAS was highly similar to that observed by LAB-TRFLP, with a diverse early mixture of *Leuconostoc spp.*, *Lactococcus*, *Streptococcus*, and *Lactobacilli*, quickly dominated by *Pediococcus* within the first few weeks. BAS data suggest, however, that *Pediococcus* was abundant at wk 0. These data also indicate a large population of “other *Lactobacillaceae*” dominant at wk 2 and 98; this OTU (at this and other timepoints) most likely represents an amalgam of *Pediococcus* and the minor LAB observed at other time points grouped into one OTU due to sequencing error resulting in shorter sequences following quality filtration, not a distinct population. Notably, BAS was unable to differentiate LAB below the species level, so we must likewise rely on LAB-TRFLP to show species-level changes.

Two major advantages of BAS over TRFLP are that OTU identification is actually sequence-based and that sequencing is much more sensitive than TRFLP (which relies on detection of fluorescent labels), facilitating the identification of minor populations (such as the unidentified Firmicutes detected by TRFLP). One of the most abundant minor populations was *Alphaproteobacteria* (as much as 7%), including *Acetobacter* and *Brevundimonas*; the *Betaproteobacteria Ralstonia* and *Comamonadaceae* were also detected in low abundance, as were *Actinomycetales*, corroborating TRFLP data. While enterobacteria certainly represent a major population, emerging minor populations of other *Gammaproteobacteria* were also detected in wk 99–173, including *Acinetobacter* (as high as 1%, at wk 148), *Halomonas*, and *Pseudomonas*. These bacteria were detected after rigorous OTU filtration was applied, thus all represent >0.01% of total sequence abundance. A complete list of OTUs detected above this threshold, as well as relative abundance by sample, is presented in Table S2.

### Batch-to-batch Consistency

**Figure 5:**
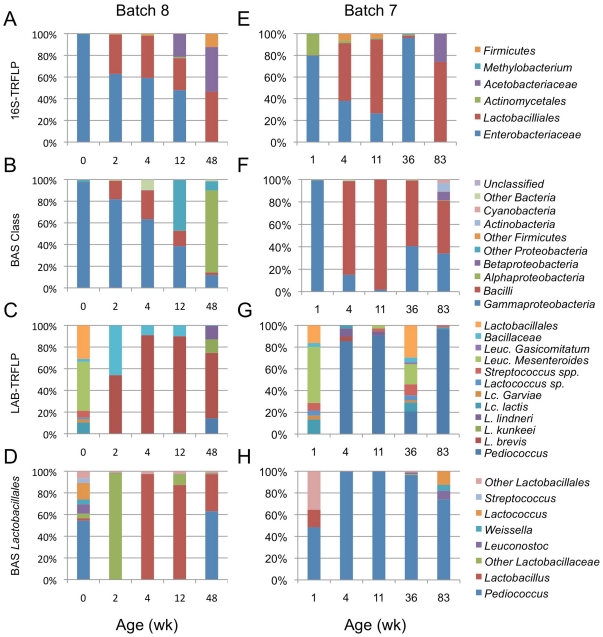
Parallel ACA batch comparison. Comparison of universal bacterial community structure (A,B,E,F) and *Lactobacillales* (C,D,G,H) in Spring batch 7 (*right*) and Winter batch 8 (*left*). *Panel A/E*: Relative abundance of 16S-TRFLP bacterial OTUs. *Panel B/F*: Class-level abundance per sample as percentage of total BAS sequences. *Panel C/G*: Relative abundance of LAB-TRFLP OTUs. *Panel D/H*: Genus-level relative abundance of *Lactobacillales* BAS sequences. All TRFLP samples are averages of duplicate samples. *y*-axes indicate relative OTU abundance.

Most batches exhibited consistent microbial transitions involving the same bacterial and yeast taxa. One exception was batch eight, for which LAB-TRFLP revealed batch-to-batch inconsistency not observed with 16S-TRFLP. Figure 5 compares batches seven (representative of the typical profile observed across batches) and eight using 16S-TRFLP (A,E), Class-level BAS (B,F), LAB-TRFLP (C,G), and genus-level BAS data for *Lactobacillales* (D,H). Class-level structure (Illumina) and resolution to family level (16S-TRFLP) suggest very close correspondence of these batches, with an initial dominance of enterobacteria quickly succeeded by *Lactobacillales*, the only dramatic observable differences being in minor OTUs (Figure 5A,B,E,F). However, LAB-TRFLP demonstrates a rapid divergence: whereas wk 0 (batch 8) and wk 1 (batch 7) showed very similar profiles (primary *Leuconostoc spp.*, as described above), by wk 2 (batch 8) and wk 4 (batch 7)—and through the remainder of the observation period—batch 7 was dominated by *Pediococcus* (also the dominant LAB in batches 1–6) and batch 8 was dominated by *L. brevis*. Beyond *Lactobacillales*, however, this batch demonstrated typical profiles for all other taxa, as shown by BAS data (increased levels of *Proteobacteria* correspond to *Acetobacteraceae*; Figure 5).

### Beta-diversity

Principal coordinates analysis (PCoA) was used to visualize relationships among samples and OTUs (as loadings) in a two- or three-dimensional space, in order to reveal underlying trends in these time-based data. Principal coordinates (PC) were calculated from both TRFLP and BAS data for two separate purposes: non-phylogenetic (Bray-Curtis) beta-diversity analysis using TRFLP to compare diversity across the complete dataset (as Illumina only represents a subsample), and weighted UniFrac clustering based on BAS of a representative subset to determine whether microbial community differences among samples are phylogenetically significant. Non-phylogenetic, count-based beta-diversity measures (such as Bray-Curtis) assume equidistant similarity among OTUs (representing a “star phylogeny”) [23], so true phylogenetic measures are extremely useful (when available) to determine whether communities are not only structurally distinct but also represent significant evolutionary diversity [23].

**Figure 6:**
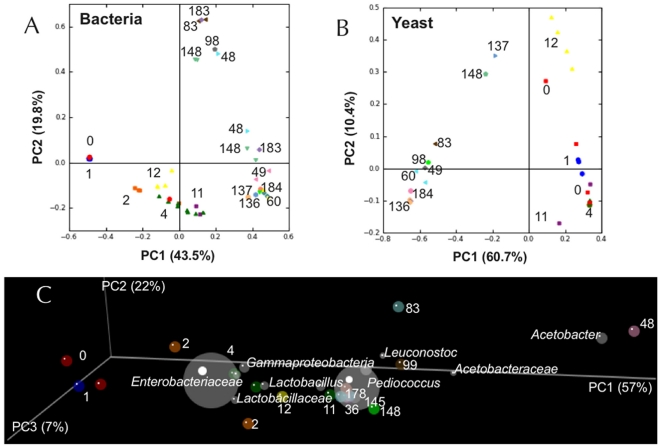
Principal coordinates analysis of ACA microbial succession. PCoA of Bray-Curtis dissimilarity scores derived from 16S-TRFLP of universal bacterial communities (*Panel A*) and ITS-TRFLP of yeasts (*Panel B*). Samples are colored by age (wk), as indicated by adjacent number. Sample distance is a function of shared OTU similarity. *Panel C*: 3-dimensional PCoA of weighted UniFrac distance of BAS of ACA samples. Samples are colored by age (wk), as indicated by adjacent number, their distance within the 3-dimensional space being a function of the phylogenetic similarity and abundance of their constituent taxa. Grey bubbles represent correlation of loadings (as taxonomic groups) along the same coordinates; their placement explains how much variance along each PC is explained by these taxa, with size as a function of relative abundance.

PCs of Bray-Curtis dissimilarity scores derived from raw TRFLP data (prior to taxonomy assignment/grouping) were calculated to compare OTU diversity among samples at different time points. PCoA of both 16S-TRFLP (Figure 6A) and ITS-TRFLP (Figure 6B) data reveal time-based clustering of samples. For both bacterial and yeast communities, the progression from early to late-fermentation is strongly correlated with the first PC (explaining 43.5% and 60.7% variance, respectively), whereas the second PC primarily explains variance among late-fermentation samples (accounting for 19.8% and 10.4% of the variance, respectively). PCoA of Bray-Curtis scores calculated from LAB-TRFLP data demonstrates clustering related to batch but not to age (except at the individual-batch level, data not shown), due to batch-to-batch variation in dominant LAB. PCoA of weighted UniFrac distance (Figure 6C) reveals a similar relationship among samples, with a time-based progression strongly correlated with the first PC (57%). PCoA loadings (as OTUs), represented by grey bubbles, are plotted to demonstrate which OTUs best describe sample variance along PCs. The first PC is positively correlated with *Lactobacillales* and *Acetobacteraceae* and negatively with *Enterobacteriaceae*, explaining the majority of phylogenetic variance observed among samples. The second PC explains much less variance (22%), probably because the minor OTUs accounting for spread among Bray-Curtis dissimilarity scores for late-fermentation samples are phylogenetically related to the temporally dominant microbiota, to the effect that UniFrac concentrates and focuses sample variation around the core taxonomic shift from *Enterobacteriaceae* to *Lactobacillales*. Greater deviation from the age-based trend is seen with UniFrac clustering, as samples displaying a bloom of *Acetobaceraceae* correlate more positively with the first PC, removing from the trend and compressing the variance otherwise explained by the core taxonomic shift.

## Discussion

Culture-independent, molecular techniques for community profiling have not been used previously to study mixed-microbial beer fermentations, including lambic-style beers. The microbial ecology of lambic, the Belgian predecessor of ACA, has only been characterized using traditional, culture-dependent techniques [1], [24] paired with extensive chemical analyses [3], [4], [25], setting the foundations of current knowledge on spontaneous beer fermentation phenomena. Therefore, this study utilizing TRFLP and BAS for community profiling of ACA fermentations was useful not only for characterizing the complete microbiota of this previously uncharacterized fermentation, but also for updating our current knowledge of spontaneous beer fermentation ecology.

Viewed at higher taxanomic levels (e.g., judging from 16S-TRFLP alone), the microbial succession of ACA fermentation appears to closely parallel that previously observed in lambic [1], [24]: a brief, early bloom of enterobacteria before LAB and *Saccharomyces* conduct the main fermentation, and finally domination by *Brettanomyces* during the long maturation period. However, lower-level taxa reveal subtle difference between these fermentations. A different set of enterobacteria are involved, with ACA primarily dominated by *Klebsiella* spp., as well as *Enterobacter* spp., *P. carotovorum*, and *S. ureilytica*, as compared to lambic, dominated by *Enterobacter*, *Hafnia*, *Escherichia*, and *Citrobacter* [24]. Whereas *Pediococcus* spp. were the only LAB cultured during lambic fermentation [1], ACA involves several lactobacilli as well as *Leuconostoc*, *Lactococcus*, and a range of other LAB (though pediococci dominated most batches). Finally, BAS uncovered several minor populations of *Alphaproteobacteria*, *Gammaproteobacteria*, and *Actinomycetales*, most of which have never before been detected in beer, most likely as molecular community profiling methods have never been used to analyze beer fermentations. Likewise, ACA fermentation involves a diverse set of minor yeasts, including *Rhodotorula*, *Cryptococcus*, *Pichia*, and *Candida*, many of which have not been described previously in beer, let alone lambic.

This core microbial succession in ACA closely follows a pH/nutrient gradient, with the initial explosive growth of *Enterobacteriaceae* curtailed by a rapid pH drop in the first 8 wk, decreasing overall diversity and selecting primarily for *Lactobacillaceae* for the remainder of fermentation. *Saccharomyces* also follow the decline of enterobacteria, possibly due to relief from competition and acclimation to inhibitory factors such as carboxylic acids produced by enterobacteria and oxidative yeasts [26], [27]. Between wk 2–9, the majority of sugars (as °Plato) were consumed, reaching ∼80% attenuation (roughly the amount of *Saccharomyces*-fermentable sugars contained in an all-malt wort). During this time *Saccharomyces* were the only yeast detected by ITS-TRFLP and total yeast populations were 100-fold those at both wk 0 and 11. The rapid drop in yeast counts and disappearance of *Saccharomyces* quickly follows this point, most illustrative of *Saccharomyces* flocculation following full attenuation, as is typical for beer fermentation. This niche is in turn filled by *Brettanomyces*, which gradually superattenuates the beer (seen as °Plato reduction beyond 20%) during the remainder of the fermentation. This gradient can also be observed via clustering of samples based on UniFrac distance or Bray-Curtis dissimilarity, with the most dramatic shifts in correlation along the first PC (i.e., diversity turnover) occurring at these stages (pH drop and full attenuation).

The core microbial profile observed in ACA appears highly conserved at the taxonomic depth achieved here, in spite of the fact that no inoculum is ever introduced to ACA wort. This includes the rare microbiota detected by BAS, the domination of *L. brevis* in one batch detected by LAB-TRFLP being the only considerable deviation. While reused barrels are an obvious source of microbial carryover, barrels for ACA production are sourced from diverse locations, including spirits production, so cannot be identified as the sole source, and even unused barrels led to replication of the microbial profile (data not shown). Enterobacteria are known regular contaminants of wort in typical brewhouse operations [26] and their ubiquity would make them difficult to eliminate from ACA when conducting spontaneous fermentation. Likewise, LAB and yeasts are common brewery microbiota, so could be introduced by surface exposure during transfer or on airborne particulates. However, the fact that the same lower taxa and succession dynamics are observed suggests the involvement of a stable brewhouse microbiota, as well as the general selective pressure exerted during fermentation (pH drop, nutrient sequestration).

This stable population may hint at the existence of “microbial terroir,” or the establishment of stable, site-specific microbiota potentially impacting product quality criteria of beverage fermentations. Chances are species of *Saccharomyces* and *Brettanomyces* become enriched and established within the brewery environment, as shown in lambic breweries in Belgium [2], resulting in a semi-consistent inoculation as these yeasts are introduced from the brewery environment during coolship exposure, much in the same way that spontaneously fermented wine most likely becomes inoculated by *Saccharomyces* spp. enriched on winery equipment surfaces [27], [28], [29], [30]. In all of these spontaneously fermented beverages, such enrichment is likely a saving grace, ensuring a certain degree of fermentation consistency as competently fermentative, competitive yeasts dominate early in the fermentation, exerting selective pressure for ethanol- and pH-tolerant microbes. This is particularly pertinent to spontaneously fermented beers, as the wort is sterilized by boiling and thus would rely entirely on environmentally introduced and barrel-associated microorganisms to conduct the fermentation. As the ACA studied in this work is a seasonal product of a full-scale-production craft brewery, unlike the dedicated lambic breweries of Belgium, the house strains of *S. cerevisiae* are likely heavily enriched in this environment, resulting in the detected dominance of this yeast in these fermentations from wk 0, already exerting enough pressure to select for fairly consistent community profiles batch-to-batch. This concept of microbial terroir must be followed up in future studies comparing ACA microbial succession at the strain level at multiple sites to fully assess the sources, consistency, and quality impact of ACA microbiota.

Surprisingly, BAS did not dramatically increase the taxonomic depth beyond that achieved by TRFLP for the dominant microbiota. Neither 16S-TRFLP nor deep sequencing could delve below family-level assignment of enterobacteria, and while sequencing did identify genera of *Lactobacillales* in many cases, LAB-TRFLP could identify most populations to species with greater reliability than sequencing, so was used to improve resolution in both cases. This may be partially explained by two reasons: 1) sequencing-error-truncated reads resulted in large gaps of OTUs only identifiable as *Lactobacillaceae*; and 2) at the time of writing, paired-end reads are currently not supported in QIIME, so paired-end reads are analyzed and interpreted as separate, shorter single-end reads. How much taxonomic acumen would be added by the extra 100 nt of a full, concatenated V4 read (∼250 nt) is yet to be determined and is likely regionally dependent. What BAS did improve is detection and identification of the rare microbiota of ACA, the detection limits (fluorescent peak-to-noise ratios) and taxonomic assignment (multiple restriction fragment comparison) of TRFLP being inadequate to identify most OTUs representing <1% of the community. However, BAS is still limited for deep exploration of rare taxa due to the lack of a codified quality filtration strategy [15], and OTUs currently require rigorous filtration to confidently differentiate real organisms from erroneous OTUs. Thus, some minor OTUs detected in ACA by TRFLP, such as *Methylobacterium* and some *Lactobacillales*, were detected below 0.01% relative sequence abundance and consequently removed, leading to an underestimate of true sample diversity. Advancements in sequencing technology continue to improve gigabase-per-run recovery—increasing multiplexing capacity and decreasing cost per sample—and error rates—permitting greater read length and post-filtration recovery of longer, high-quality sequences—providing hope that these limitations will be resolved in the near future. These improvements and reductions in cost per-sample are bringing NGS technologies within grasp of more researchers, promising larger-scale, higher-resolution, multivariate studies of fermented food systems utilizing the rare-microbiome exploration and phylogeny-based comparisons provided by these new tools.

## Supporting Information

Figure S1 Detailed schedule for representative mosaic sampling across 3 yr of ACA fermentation. In order to collect samples representing 3 yr of ACA fermentation, overlapping batches were tested in replicate barrels. Bubbles represent analyses performed on individual samples from individual batches and barrel replicates. Red, 16S-TRFLP; Green, ITS-TRFLP; Blue, LAB-TRFLP; Yellow, BAS; Black, cultured; Cyan, QPCR.
(TIFF)

Figure S2 Alpha rarefaction of barcoded sequencing operational taxonomic units. Observed species alpha-rarefaction calculated using QIIME.
(TIFF)

Table S1 BAS primers and barcodes used in this study.
(DOC)

Table S2 Genus-level assignment of OTUs detected by BAS. Values correspond to relative sequence abundance represented by that OTU for that sample. Only OTUs representing >0.01% sequence abundance are shown.
(TXT)
