# Supplementary figures and images for: Brewhouse-Resident Microbiota Are Responsible for Multi-Stage Fermentation of American Coolship Ale
Source: PLoS One. 2012 Apr 18;7(4):e35507. doi: 10.1371/journal.pone.0035507 (PMC3329477; doi:10.1371/journal.pone.0035507)

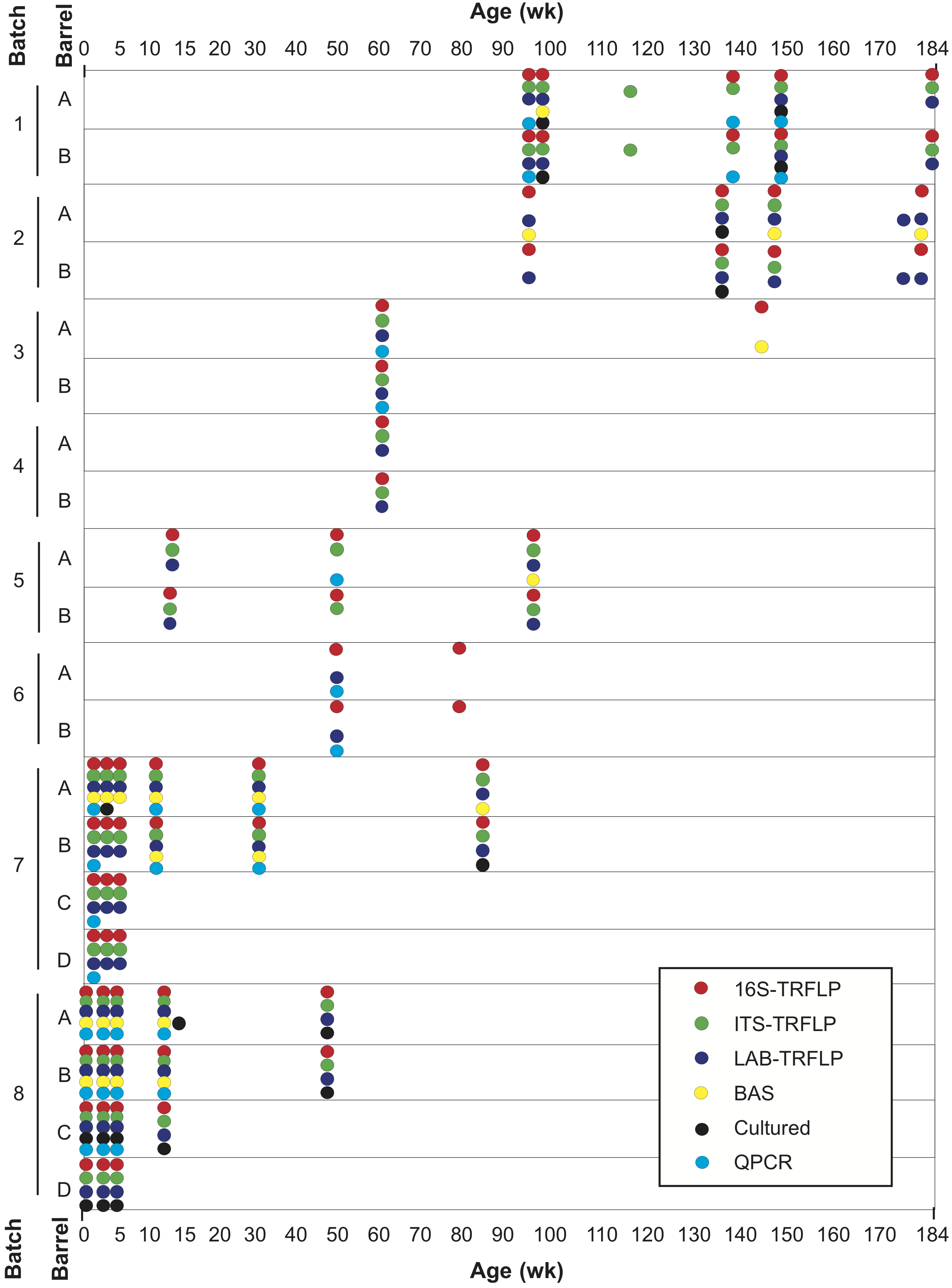

Supplement: Figure S1 — Detailed schedule for representative mosaic sampling across 3 yr of ACA fermentation. In order to collect samples representing 3 yr of ACA fermentation, overlapping batches were tested in replicate barrels. Bubbles represent analyses performed on individual samples from individual batches and barrel replicates. Red, 16S-TRFLP; Green, ITS-TRFLP; Blue, LAB-TRFLP; Yellow, BAS; Black, cultured; Cyan, QPCR. (TIFF) [file pone.0035507.s001.tif]

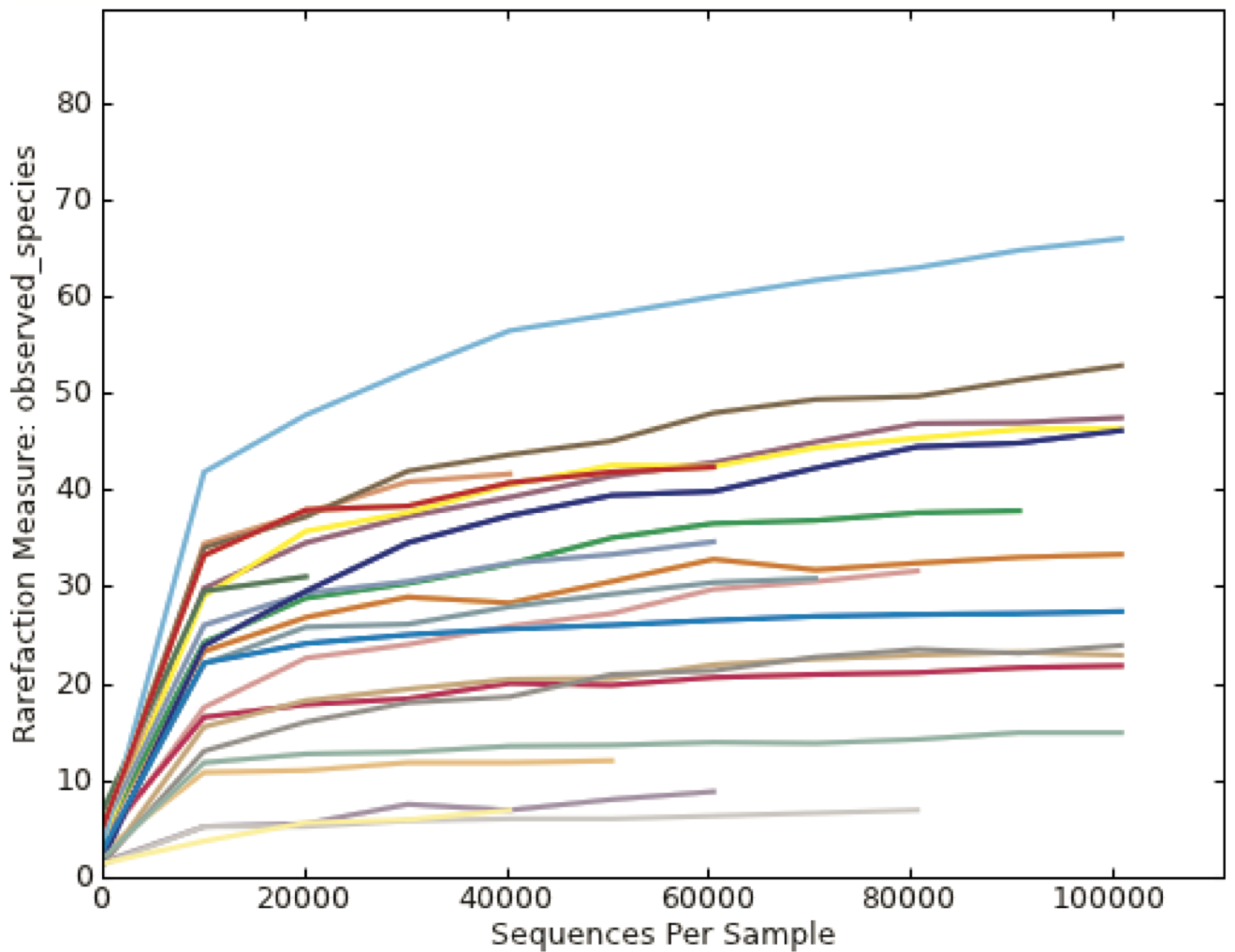

Supplement: Figure S2 — Alpha rarefaction of barcoded sequencing operational taxonomic units. Observed species alpha-rarefaction calculated using QIIME. (TIFF) [file pone.0035507.s002.tif]
