# Supplementary material for: Brewhouse-Resident Microbiota Are Responsible for Multi-Stage Fermentation of American Coolship Ale
Source: PLoS One. 2012 Apr 18;7(4):e35507. doi: 10.1371/journal.pone.0035507 (PMC3329477; doi:10.1371/journal.pone.0035507)
Supplement: Table S1 — BAS primers and barcodes used in this study. (DOC) [file pone.0035507.s003.doc]

**Table S1.** BAS primers and barcodes used in this study.

Underlined region indicates Illumina adapter sequence. Bold-face text indicates PCR primer region, preceded by linker sequence. Poly-N string in forward primer denote barcode sequence.

Forward Primer:

AATGATACGGCGACCACCGAGATCTACACTCTTTCCCTACACGACGCTCTTCCGATCTNNNNNNNNGT**GTGCCAGCMGCCGCGGTAA**

Reverse Primer:

CAAGCAGAAGACGGCATACGAGATCGGTCTCGGCATTCCTGCTGAACCGCTCTTCCGATCTCC**GGACTACHVGGGTWTCTAAT**

Barcodes used in this study:

AAGCTTGC

ACAGAGAC

AGCTACGT

AACCGCTA

AACCGGAA

AACGAAGC

AACGATCC

ACACGTGT

ACAGCAGA

ACAGCTCA

AACCAAGG

AACCATGC

AACCTTCC

AACGATGG

AAGCTAGG

AAGCTTCG

AAGGATGC

AAGGCCTT

AAGGCGTA
